# Measuring the Operating Condition of Induction Motor Using High-Sensitivity Magnetic Sensor

**DOI:** 10.3390/s25144471

**Published:** 2025-07-18

**Authors:** Akane Kobayashi, Kenji Nakamura, Takahito Ono

**Affiliations:** 1 Department of Mechanical Systems Engineering, Tohoku University, Sendai 980-8579, Japan; 2 Department of Management Science and Technology, Tohoku University, Sendai 980-8579, Japan; kenji.nakamura@tohoku.ac.jp; 3 Micro System Integration Center (µSiC), Tohoku University, Sendai 980-8579, Japan

**Keywords:** induction motors, magnetic sensor, non-contact monitoring, torque, power

## Abstract

This study aimed to monitor the operating state of an induction motor, a type of electromagnetic motor, using a highly sensitive magnetic sensor, which could be applied for anomaly detection in the future. Monitoring the health of electromagnetic motors is very important to minimize losses due to failures. Detecting anomalies using the changes compared with the initial state is a possible solution, but there are issues such as a lack of training data for machine learning and the need to install multiple sensors. Therefore, an attempt was made to acquire the various operating states of a motor from magnetic signals using a single magnetic sensor capable of non-contact measurement. The relationships between the magnetic flux density from the motor and the other motor conditions were investigated. As a result, the magnetic spectrum was found to contain information on the rotor rotation frequency, torque, and output power. Therefore, the magnetic sensor can be applied to monitor a motor’s operating conditions, making it a useful tool for advanced data analysis.

## 1. Introduction

In modern society, motors and magnetically driven machinery are key elements in powering many sectors, including industrial machinery, household appliances, and transportation [1]. Motors are essential in manufacturing and everyday life as they improve production efficiency and enable automation. Their failure can lead to production line stoppages and severe damage; thus, preventive maintenance and fault diagnosis are essential.

Vibration analysis, temperature monitoring, and encoders are used to monitor motor status and diagnose faults. For example, it has been reported that signals from acoustic and vibration sensors can be learned by convolutional neural networks [2], and current and vibration sensor data can be compared with the initial state for short circuits in brushless DC motor windings to classify the fault class [3]. Using these methods, internal motor wear and abnormalities can be detected at an early stage, and the time for repair or replacement can be determined. Fault diagnosis contributes to increasing the uptime of motors and reducing costs.

There are various methods of sensing motors, each with their own characteristics. Sensors for measuring mechanical vibration, such as accelerometers, can predict shaft distortion, bearing deterioration, etc. [4,5,6]. Temperature sensors can also be used to detect abnormal heat rises and prevent damage due to overheating. However, they can only detect abnormalities during heat generation and cannot be used for preventive diagnostics. Accurate temperature measurement is essential for the safe operation of permanent magnet synchronous motors, and research on predicting temperature using machine learning has been conducted [7]. The current sensors are widely used in general and can diagnose a wide range of electrical and mechanical anomalies using changes in current [8]. However, they require specialized analysis and are susceptible to false positives. Complete monitoring of a motor’s condition requires the use of several sensors, and even fault diagnosis requires a combination of multiple methods for accurate diagnosis [9].

Magnetic sensors detect variations in the magnetic field from a motor and may be used to provide information on the operating conditions and internal faults of a motor. The magnetic field inside a motor varies depending on the current supplied to the motor and the state of the rotor. Regular operation shows a specific pattern of magnetic field variations, but this pattern changes when a fault or abnormality occurs. Magnetic sensors have the advantage of non-contact measurement, without directly touching the motor. Recent magnetic sensors have a compact size and are highly sensitive [10,11,12,13,14]. In addition, magnetic sensors have the potential to address multiple issues, such as winding shorts, rotational imbalances, and current anomalies. For example, short-circuit fault detection in permanent magnet synchronous motors has been conducted using machine learning with magnetic field sensors [15]. Similarly, demagnetization faults in permanent magnet synchronous motors under steady-speed conditions have been diagnosed [16,17]. The defect patterns in the leakage magnetic flux spectrum of permanent magnet synchronous motors have been shown to provide excellent results for the diagnosis of magnet faults [18].

While many studies have focused on monitoring techniques for DC motors, there has also been significant research on monitoring the condition of induction motors. The strain of the magneto-strictive material in induction motors is measured using fiber Bragg grating (FBG) strain sensors, and a method for monitoring the condition of induction motors and measuring operating conditions was proposed [19].

It has been demonstrated that combining current and stray flux analyses enables the effective detection of rotor faults in soft-started induction motors through spectral features and machine learning algorithms [20]. A previous study introduced a non-invasive method for monitoring induction motor loads using giant magnetoresistance (GMR) sensors to detect stray magnetic flux and analyze time–frequency spectrograms during both transient and steady-state operation [21]. That work demonstrated how variations in stray flux indicated changes in load and mechanical faults, particularly during motor startup. In contrast, our study employs a high-sensitivity magneto-impedance (MI) sensor to quantitatively determine the torque, rotor speed, and output power from the spectral features in the leakage magnetic flux. While the earlier approach focused on pattern-based signal analysis, this work establishes direct physical relationships between the spectral characteristics and motor operating parameters, enabling simple and model-free monitoring using a single sensor [22]. A comparative study of induction motor monitoring techniques shows the strengths and limitations of vibration, current, and stray flux sensing methods for fault detection across various conditions. Unlike such multi-sensor approaches, our study uses a single high-sensitivity MI sensor to quantitatively estimate torque, rotor speed, and power, based on their direct physical correlations with spectral features.

This work introduces a single high-sensitivity magneto-impedance sensor that quantitatively determines the torque, rotor speed, and output power from the spectral characteristics of the leakage flux. By establishing explicit physical relationships between spectral peaks, slip, and mechanical load, the method eliminates the need for additional current, vibration, or torque sensors, yielding a compact, model-free platform for the real-time condition monitoring of the induction motors used in pumps, compressors, fans, and electric vehicles. This paper investigates those relationships experimentally and provides the necessary basis for future fault analysis frameworks.

## 2. Materials and Methods

A. Induction motor and working principle

A single-phase squirrel-cage induction motor (Oriental Motor Co., Ltd., Tokyo, Japan, 0IK1A-AW2J) was used as the test motor. A relatively small motor was chosen to validate the concept in a controlled and low-power setup, which minimized safety risks and allowed flexible testing. We believe that similar results would be obtained for induction motors of different sizes, as long as the motor operates on the same fundamental electromagnetic principles. The structure of the induction motor used in this experiment is shown in the Supplementary Materials, Figure S1. The stator was wound with eight coils, arranged vertically, horizontally, and diagonally, producing a magnetic field with two pairs of poles.

In an induction motor, the three-phase currents applied to the stator windings create a rotating magnetic field whose synchronous speed is governed by the supply frequency and the number of pole pairs. This traveling field induces electromotive forces in the rotor conductors; the resulting currents interact with the stator field to generate torque, so the spatial and temporal distribution of the magnetic flux density **B** in the air gap is a primary determinant of motor performance. In an ideal machine, the air gap permeance is uniform, and **B** is a pure space and time sinusoid. However, practical considerations such as slotting, winding layout, and core nonlinearities introduce distortions, as explained next [23].

**Slotting effects**: The axial slots in the stator and rotor periodically modulate the air gap permeance, producing spatial harmonics in the magnetic flux density. These harmonics, typically the 5th, 7th, or higher orders, distort the ideal sinusoidal distribution and appear as sidebands in the spatial spectrum.

**Magnetic saturation**: When parts of the core approach saturation, the nonlinear B–H response causes the magnetic flux to deviate from a pure sinusoid, generating time-domain harmonics at multiples of the supply frequency. This reflects the intrinsic nonlinearity of the magnetic circuit.

**Slip-dependent variation**: Rotor slip shifts the frequency of the flux components observed in the air gap. As the slip changes with the load, the spectral content of the flux density dynamically modulates, reflecting variations in motor speed and torque.

Nonlinear flux components appear in the spectrum as higher-order harmonics, such as the 3rd, 5th, and 7th, reflecting deviations from ideal sinusoidal behavior due to structural and magnetic non-idealities. These harmonics can modulate torque output and contribute to vibration, especially under low-speed or fluctuating loads. Importantly, their spectral features serve as diagnostic indicators—changes in harmonic patterns may signal rotor asymmetry, eccentricity, or insulation degradation.

In practice, the ideal single-frequency flux waveform is rarely observed. Slotting, magnetic saturation, and slip introduce systematic nonlinearities, and the resulting spectral harmonics offer valuable insights into motor condition.

B. Magnetic Sensor

There are various types of magnetic sensors, but, in this experiment, a single-axis MI (magneto-impedance) sensor (Aichi Steel Corp., Aichi, Japan, MI-CB-1DH-S-A) based on the magnetic impedance effect was used [24]. The typical sensitivity of the sensor was approximately 30 mV/Oe, with a noise floor in the range of 100–200 pT/√Hz. TMR and GMR sensors can achieve sub-nT resolution, their linear measurement range often remains limited to tens or hundreds of µT, and sensitivity degrades or saturation occurs at higher fields [25]. In contrast, MI sensors maintain high sensitivity in the nT range while offering a markedly wider dynamic range (up to ± tens of mT), making them better suited for applications like motor condition monitoring, where both weak leakage flux and stronger transient fields must be captured. The power line supplying current to the motor also generated a 50 Hz magnetic field. To minimize the influence of 50 Hz magnetic fields, the magnetic sensor was installed away from the power supply line, and the cable was secured to prevent movement.

C. Current Sensor, Toque sensor, Optical rotation sensor, Data acquisition

The currents supplied to the motor were measured by current sensors (QingxianZeming Langxi Electronic Device Co., Ltd., Nanjing, China, ZMCT103C). The torque applied to the motor was measured by a laboratory-made torque sensor with a load cell. The end of the L-shaped load cell with strain sensors was pressed against the shaft, and torque was applied to the shaft. The frictional force generated a load torque on the shaft, and the magnitude of this force *F* was measured at the same time. The magnitude of the torque was varied by changing the horizontal force acting between the shaft and the L-shaped end of the load cell. The radius *r* of the shaft was 5 mm. Therefore, the torque *τ* was given by *τ = rF.*

The rotation of the rotor was also measured by a laboratory-made optical rotation sensor. One part of the shaft was colored with a black marker, which was irradiated with an LED, the intensity of the reflected light was measured with a photodiode, and the rotational frequency of the rotor was measured from the intensity variation.

The outputs of the magnetic sensor, the three current sensors, the photodiode, and the torque sensor were connected to an AD converter (Measurement Computing Co., Ltd., Shanghai, China, USB-1608FS-Plus), and data for comparison of the sensor outputs were acquired at the same time.

**Figure 1:**
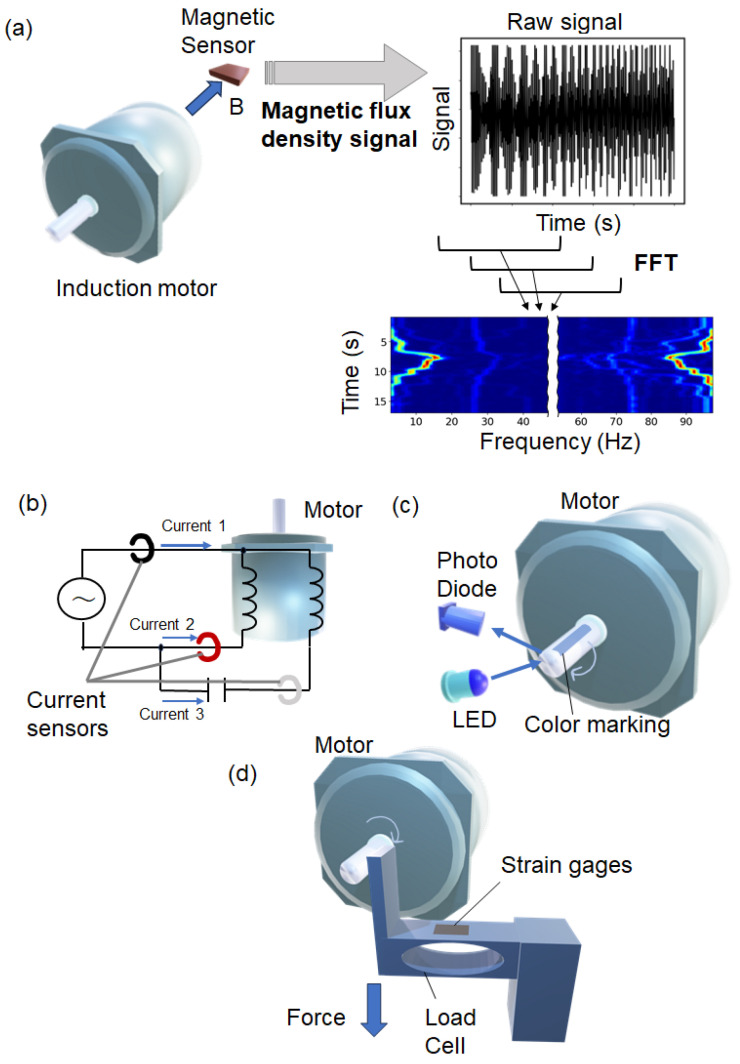
Schematic of various measurements. (**a**) Measurement of the magnetic flux density from the motor using magnetic sensor. The signals are Fourier-transformed, and a frequency versus time map is obtained, where color shows the intensity of the signal. (**b**) Current measurement of three wires using three current sensors. (**c**) Rotation speed measurement using an optical sensor. (**d**) Torque application and torque measurements.

The raw data of each component were measured as shown in Figure 1. Short-time Fourier transform spectra were obtained by performing Fourier transforms on each raw data segment of the same size. The sampling frequency was 4000 Hz, the time difference between neighboring segments was 0.15 s, and the segment length was 1.125 s. For the Fourier transform, we used the Numpy library 2.0 in Python. The Hanning window was used for the window function. In addition, “find_peaks” from the Scipy library was used to detect the peaks of the time-varying frequency signal in the spectrum.

## 3. Results

To investigate the relationship between the leakage magnetic flux density and the operating characteristics of an inductive motor, the experimental setup shown in Figure 1 was used. The magnetic flux density was evaluated by the magnetic sensor, and the obtained signal was transformed into the frequency domain using fast Fourier transform (FFT), as shown in Figure 1a. The schematics of the measurement for the currents, the rotor frequency, and the torque are shown in Figure 1b, Figure 1c, and Figure 1d, respectively. The torque applied to the shaft was monitored, and there was supposed to be no torque loss in the motor; thus, the magnitude of the applied torque was the same as that of the generated torque. The currents of the three phases in the three wires supplying 50 Hz power to the motor were measured, and one of them passed through a capacitor to change the phase.

**Figure 2:**
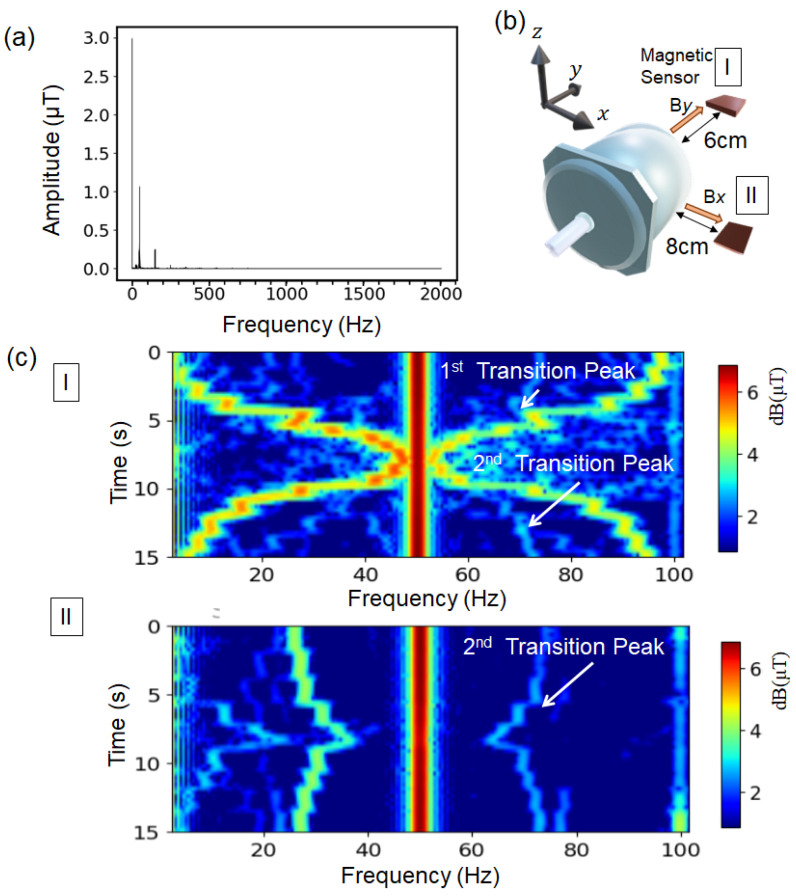
Short-time Fourier transform magnetic flux spectra depending on sensor positions. (**a**) Typical plotting spectrum of every segment. (**b**) The positioning of back (I) and side (II) of the magnetic sensor relative to the motor. (**c**) Short-time Fourier magnetic flux spectra at the back (I) and side (II) positions of the magnetic sensor. The torque load was gradually increased from time 0 and then decreased after approximately 8 s.

The magnetic sensor, were short-time Fourier-transformed while varying the load torque applied to the shaft of the motor, and the magnetic sensor output in the frequency domain was obtained over time, as shown in Figure 2a. The peaks with multiples of 50 Hz appeared, and in addition, between these fixed peaks, there were several transition peaks where the frequency varied with time in response to the load torque. As shown in Figure 2b, the magnetic signals in the *y*-, *x*-, and *z*-axis directions were acquired separately with the sensor placed at positions I and II. The optimal sensor placement was determined empirically by evaluating the signal-to-noise ratio of the magnetic flux leakage along each axis. Figure 2c shows the typical color-coded spectra of the magnetic signal for each period, overlaid and plotted on a two-dimensional graph. In this graph, the vertical axis represents the time, the horizontal axis represents the frequency, and the color indicates the amplitude of the magnetic signal. A strong magnetic flux originating from the input power was observed at a frequency of 50 Hz. The low-frequency noise components below 3 Hz from laboratory noise were excluded. Several transition peaks with varying frequencies depending on the torque were observed. Those peaks were symmetrical in the high-frequency and low-frequency regions with respect to the magnetic signal from a 50 Hz supply current frequency.

**Figure 3:**
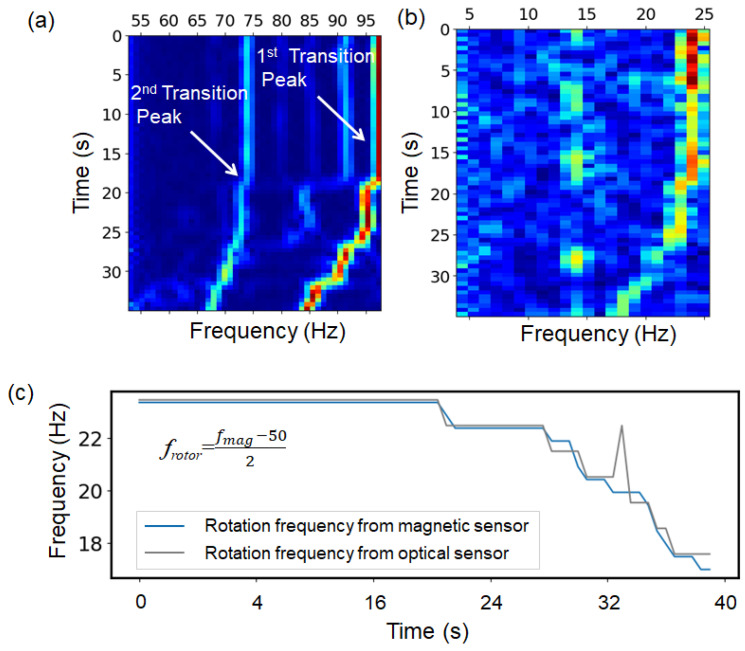
Simultaneously obtained short-time Fourier spectra of magnetic sensor and photo diode data, and the comparison of rotational frequency and converted magnetic peak frequency. Signal intensity is visualized on a 2D map using color variations. (**a**) Magnetic spectrum; (**b**) spectrum obtained by photodiode, showing rotor frequency. (**c**) Rotor speeds estimated from both spectral peaks. (Minor fluctuations in the magnetic peak intensity and calculated rotation speed may have resulted from transient mechanical vibrations and slight instability in torque sensor contact during torque application).

Figure 3a shows the time dependence of the typical magnetic flux density spectrum observed at sensor position I for frequencies *f* in the range of 50 < *f* < 100, while Figure 3b shows the simultaneously measured optical reflection spectrum. The frequency $f_{rotor}$ of the transition peak with the maximum magnitude in the light reflection spectrum was the rotational frequency of the rotor. An induced current was generated in the rotor by the magnetic fields from the stator coils, and the magnetic field also magnetized the rotor, while the rotation of the rotor induced the motion of the charges, which generated the magnetic fields. As discussed later, magnetic fields were superimposed, and different frequencies of magnetic signals were observed. Here, the frequency $f_{mag1st}$ of the maximum transition peak of the magnetic signal shown in Figure 3a was applied to the following equation, which was equal to the rotor frequency $f_{rotor}$, as given by $f_{rotor}=(f_{mag1st}-f_{e})/2$, where $f_{e}$ is the supplied power frequency ($f_{e}$ = 50 Hz). Figure 3c shows the time dependence of $f_{rotor}$ and $(f_{mag1st}-f_{e})/2$. This shows that the rotor frequency could be obtained from the transition peak frequency. This transition peak strongly appeared when the *y*-axis magnetic field was observed at sensor position I, as shown in Figure 3b. On the other hand, the relationship between the frequency of the second-largest transition peak $f_{mag2nd}$ and the rotor frequency was $f_{rotor}$ = $f_{mag2nd}$ − $f_{e}$. As shown in Figure 2c, the transition peaks were symmetrical at higher and lower frequencies relative to the 50 Hz supply power frequency, and both transition peaks were related to the rotor frequency. The rotor was affected by both the magnetic field due to the supply voltage and the rotating magnetic field that depended on the number of pole pairs *p* (*p* = 2 in this motor), which may have been the reason why two transition peaks were observed. The leakage magnetic flux density depended on the position of observation. The intensity of the main transition peak *I*_mag_1st was lower at the rear of the motor (position II) compared to the back side (position I). On the other hand, the second transition peak observed at position II was stronger than that at position I. In an induction motor, the rotating magnetic field generated by the stator induces currents in the rotor due to the relative motion (slip) between them. These induced rotor currents, in turn, generate their own magnetic fields, which interact with the stator field and produce a time-varying magnetic flux in the air gap. Because the rotor rotates slightly slower than the synchronous speed, the resulting flux observed in the stator frame continuously varies over time. This dynamic interaction is a primary source of spectral components known as transition peaks, which reflect the coupling between the rotor motion and electromagnetic induction. If the modulation is ideally symmetric, the sideband peaks on either side of the fundamental frequency are expected to exhibit equal intensity. However, structural asymmetries within the motor, such as rotor geometry, slot configuration, and non-uniformities in the air gap, can alter the coupling strength in specific directions, leading to imbalanced sideband intensities.

**Figure 4:**
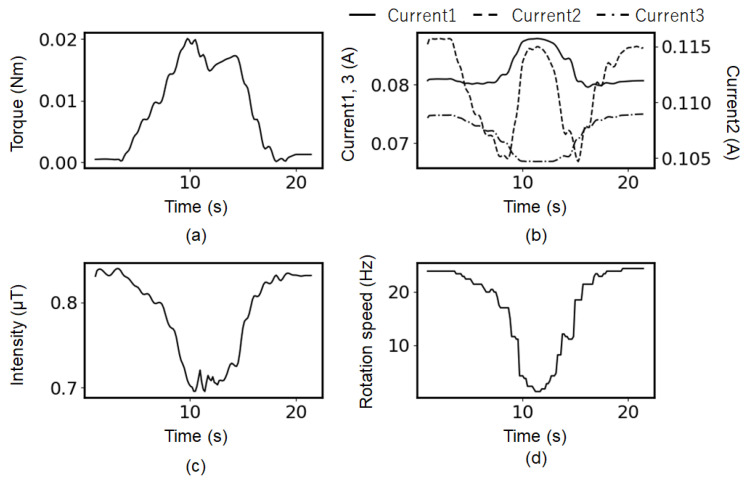
Simultaneous measurement of motor monitoring parameters. (**a**) Torque, (**b**) current, (**c**) peak intensity of 50Hz peak in magnetic flux density spectrum, (**d**) rotation speed calculated by shifting peak in magnetic flux density spectrum. (Minor fluctuations in the magnetic peak intensity and calculated rotation speed may have resulted from transient mechanical vibrations and slight instability in torque sensor contact during torque application.).

**Figure 5:**
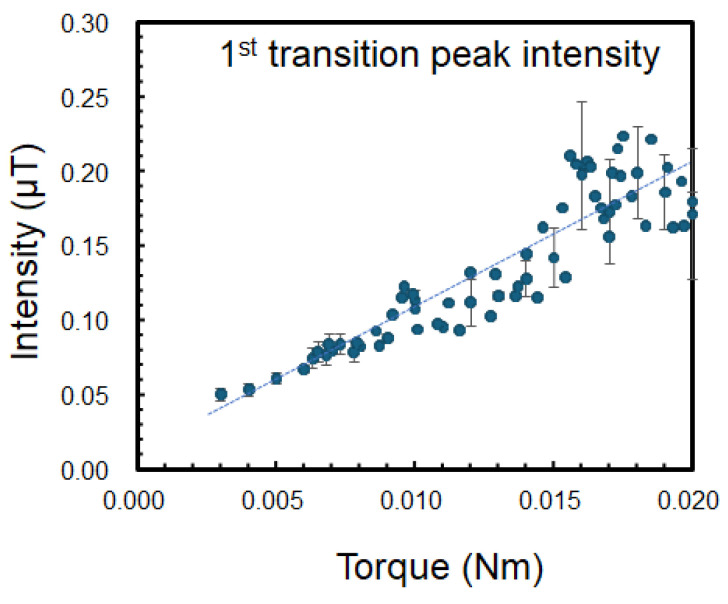
Observed relationship between magnetic flux density (Intensity) of 1st transition peak and torque.

The applied torque was varied similarly, and the time variations in each parameter were observed, as shown in Figure 4. Figure 4a shows the DC component obtained by the torque sensor (load cell). Figure 4b shows the 50 Hz component of the three currents’ spectra, and Figure 4c shows the 50 Hz component of the magnetic flux signal measured by the magnetic sensor. Figure 4d shows the rotor frequency calculated from the transition peak in the magnetic spectrum. Although the changes in the current detected by the current sensors appear complex, these results show a correlation between the torque, rotational speed, and magnetic field. In addition, the relationship between the peak intensity of the first transition peak *I*_mag_1st and the torque is plotted in Figure 5, which shows the linear dependence between them. This relationship can be expressed as

τ(Nm) = *a I*_mag_1st (µT)+ *b*, where *a =* 0.1, and *b =* 0.0068, which are fitting parameters determined from experimental data, and *b* is given by the background magnetic flux noise. The relatively large errors observed in the first transition peak intensity within the torque range of approximately 0.015–0.018 Nm in Figure 5 were primarily attributed to the mechanical vibration or chatter that occurred during torque generation.

## 4. Discussion

From previous experiments, transition peaks were observed at the sum or difference of the integer multiples of the supplied power frequency *f*_e_ and the integer multiples of the rotor frequency. The rotation of the induced charges due to the induction current generated by the stator coils generates a magnetic flux on the rotor. In addition, the rotor is magnetized by the magnetic field generated by the stator coils. When a magnetic material is magnetized by alternative magnetic fields of two different frequencies (e.g., *f*_e_, *f*_rotor_), the resulting magnetization generates the original frequencies *f*_e_ and *f*_rotor_, as well as the sum frequency $f_{e}+f_{rotor}$ and difference frequency $\left|f_{e}-f_{rotor}\right|$. The symmetry of the transition peaks on both sides of the 50 Hz power supply frequency is attributed to the modulation effects arising from the interaction between the rotor’s rotating magnetic field and the stator’s fixed-frequency magnetic field. Considering the number of pole pairs *p* in the motor (two in this motor), the rotor was also involved with a magnetic field of frequency *f*_e_/*p*.

In addition, soft magnetic materials consisting of rotors generally show nonlinear magnetization characteristics. This nonlinearity generates the high-frequency components of magnetic flux, 2$f_{e}$, 3$f_{e}$, ${2f}_{rotor}$, ${3f}_{rotor}$, etc. Therefore, the frequency components due to multiple linear combinations, such as ${nf}_{e}+{mf}_{rotor}$ (*m*, *n* are integers), possibly appear.

**Figure 6:**
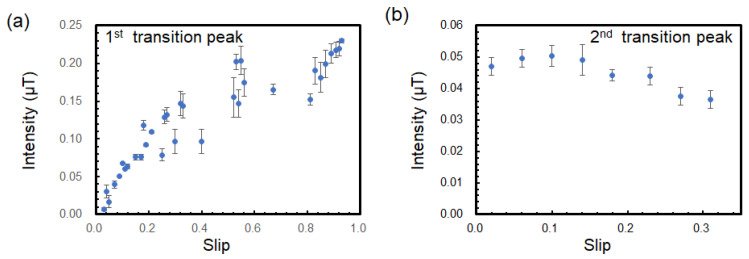
(**a**) Relationship between peak intensity of magnetic flux density of 1st transition peak and slip at sensor position I. (**b**) Relationship between peak intensity of magnetic flux density of 2nd transition peak and slip at sensor position II.

The transition peak intensity in the magnetic spectrum is considered. In an induction motor, a rotating magnetic field is generated by the currents flowing in the stator coils. This rotating magnetic field contributes to the rotor rotation, but if the rotor is perfectly synchronized with the rotating magnetic field, no induction current is generated, and no torque is produced; thereby, a slight delay (slip) is necessary. Slip *s* is a parameter calculated from the rotor frequency as given by

(1) $$s=\frac{f_{s}-f_{rotor}}{f_{s}},$$

where *f_s_* is the frequency of the rotating magnetic flux density **B_r_** at the rotor, which is given by $f_{s}=f_{e}/p$ (= 25 Hz in this study). Generally, slip is proportional to the torque τ, i.e., s∝τ. The relative velocity created by the slip causes an induced current $J\propto -\partial B_{r}/\partial t\;$ to flow in the rotor, and the interaction between this current and the rotating magnetic field generates torque $\tau \propto -\partial B_{r}/\partial t\;\times B$, where **B** is the magnetic flux density at the rotor. Therefore, the torque is possibly related to the magnitude of the transition peak. The relationship between slip and the intensity of the first transition peak when measured at sensor position I, as plotted in Figure 6a, and the relationship between slip and the intensity of the second transition peak observed at sensor position II is plotted in Figure 6b. The diagram shows some correlation between slip, torque, and the intensity of the first transition peak. When the slip increased, the intensity of the first transition peak increased. In contrast, the intensity of the second transition peak did not show a clear relationship with the torque. This result suggested that the first transition peak originated in the physical phenomenon of torque generation. The peak intensity of the 50 Hz magnetic field from the stator coil also showed a correlation with torque (Supplementary Materials, Figure S2).

**Figure 7:**
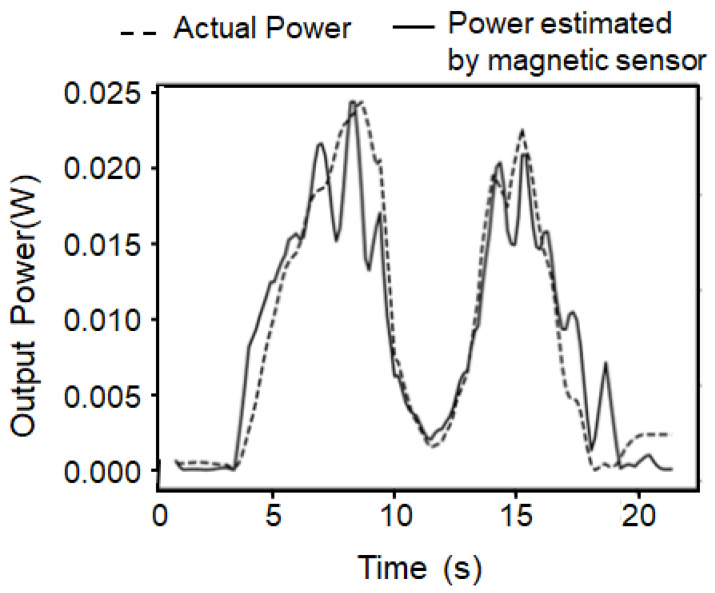
Demonstration of power monitoring of a motor using a magnetic sensor based on 1st transition peak. Applied torque was varied and the actual outputs were observed using a torque sensor and an optical rotation sensor. Using the magnetic sensor, the power was predicted from the 1st transition peak intensity and the frequency. The comparison of the two shows good correlation between them.

The output power of the motor is obtained from the torque *τ* with the load cell and the rotation frequency *f_rotor_*, which is observed by the optical sensor, as *P* = 2π*τ f_rotor_*. The torque can be estimated from the peak intensity of the first transition magnetic flux density signal, as mentioned before, and the rotor frequency can be obtained from the transition peak frequency. The actual output of the motor and the output estimated by the magnetic sensor are plotted in Figure 7. Although slight deviations are observed, the magnetic sensor enables the non-contact estimation of output power with sufficient resolution to detect changes in motor condition in real time.

Because the magnetic sensor is non-contact, needs only a small module placed near the housing, and is inexpensive enough for large-scale IoT deployment, it can continuously track motor efficiency versus output through the magnetic flux spectrum. Abnormality detection may be possible based on the magnetic flux density using big data analysis (see Supplementary Materials Figure S3). This may enable the detection of leakage currents, such as faulty insulation inside a motor. These results can form the basis for fault analysis using big data analysis or machine learning and are expected to be applied for the non-contact measurements of motor conditions and anomaly detection.

## 5. Conclusions

In this research, a high-sensitivity magnetic sensor was used to measure the operating state of an induction motor. When a short-time Fourier transform was performed on the magnetic signal, peaks that transitioned to the rotor frequency appeared, in addition to peaks at multiples of 50 Hz. The analysis of the intensity and frequency of these peaks revealed that they contained various information, including the rotational frequency, the torque, and the output power of the motor. From this information, the state of the motor at the time of an abnormality can be determined more clearly. Furthermore, these data can provide the basis for big data analysis or machine learning for the detection of motor anomalies.

## Data Availability

The data presented in this study are available on request from the corresponding author.

## Supplementary Materials

The following supporting information can be downloaded at: https://www.mdpi.com/article/10.3390/s25144471/s1. Figure S1. Single-phase induction motor used in this study and its structure. Figure S2. Relationship between torque and magnetic flux intensity for 50-Hz signal. Figure S3. Example of failure monitoring.
