# Supplementary material for: Measuring the Operating Condition of Induction Motor Using High-Sensitivity Magnetic Sensor
Source: Sensors (Basel). 2025 Jul 18;25(14):4471. doi: 10.3390/s25144471 (PMC12299784; doi:10.3390/s25144471)
Supplement: Supplementary file 1 [file sensors-25-04471-s001.zip › sensors-3730608-supplementary.pdf]

Supplementary Material

Measuring the operating condition of induction motor using high-sensitivity magnetic sensor

Akane Kobayashi, Kenji Nakamura, Takahito Ono
Graduate School of Engineering, Tohoku University, Japan.

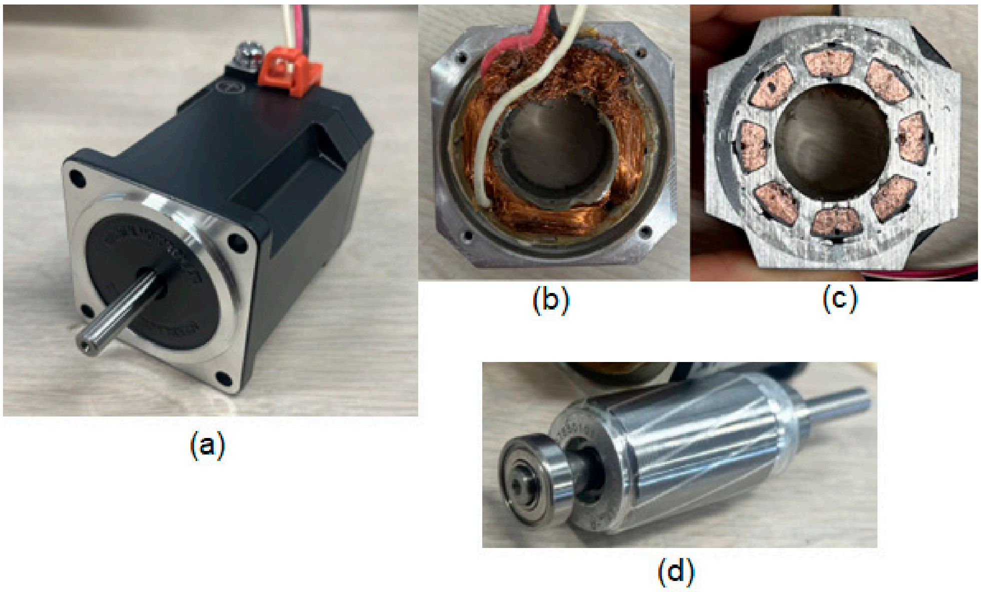

Figure S1. The single-phase induction motor used in this research and its structure. (a) The motor exterior, (b) The motor contents (Stator), (c)The cross section of the stator, (d) The rotor exterior with 20 mm diameter..

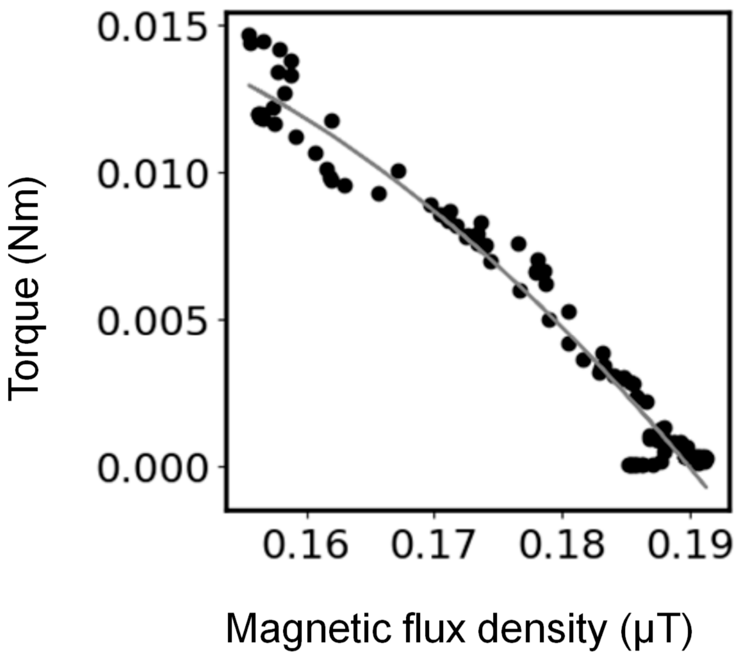

Figure S2. Relationship between the torque and magnetic flux intensity at 50 Hz signal

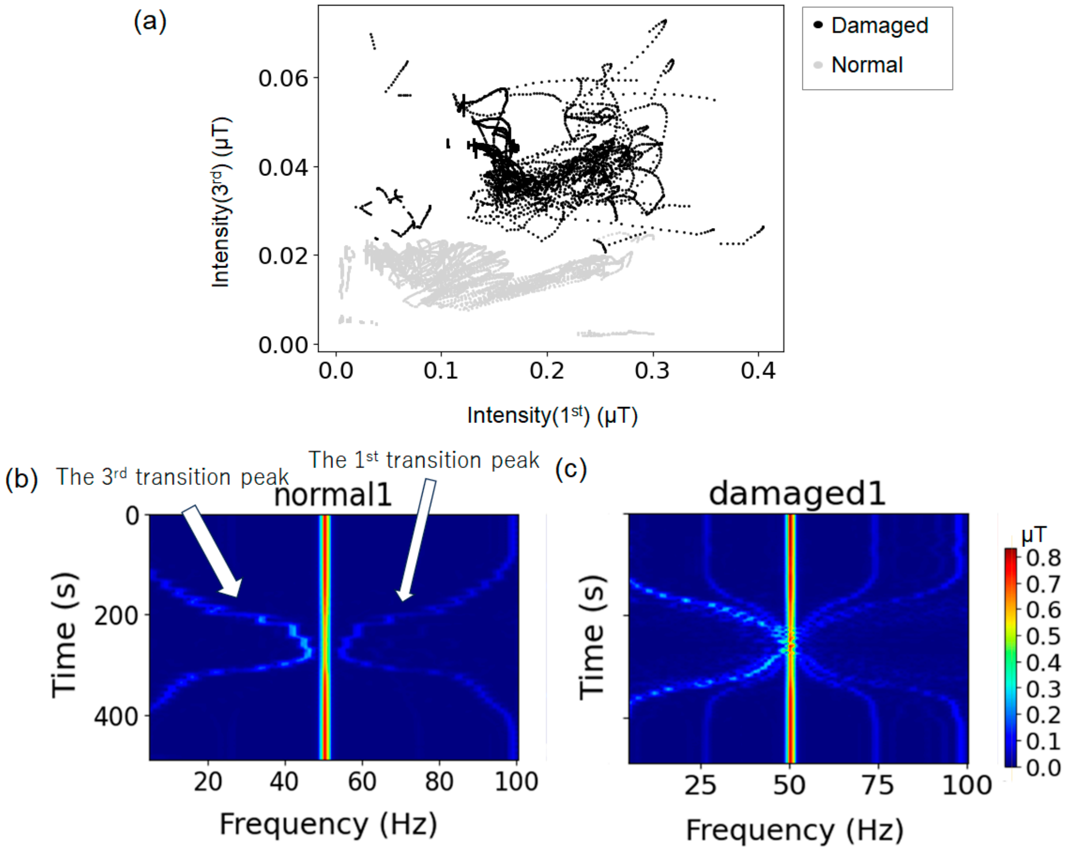

Figure S3. Example of failure monitoring. Two motors, one is the normal motor, and another is a motor with a rotor having a 1 mm-wide scratch of 0-6 mm depth and 18.5 mm length were evaluated. The intensity of the magnetic flux density peaks at varying applied torques was plotted on a 2D intensity plane, showing clear differences in the clusters between the normal and faulty motors. (a) 2D intensity plot. (b) Typical transition peak variation of the normal motor. (c) Typical transition peak variation of the failure motor.
